# “Polio resurgence in Pakistan: unraveling the causes and addressing the consequences”

**DOI:** 10.1080/20008686.2024.2447092

**Published:** 2024-12-29

**Authors:** Erum Siddiqui, Maliha Khalid, Muhammad Saad Khan, Aminath Waafira

**Affiliations:** Department of Medicine, Jinnah Sindh Medical University, Karachi, Pakistan; School of Medicine, The Maldives National University, Malé, Maldives

Poliomyelitis, often referred to as polio, is a highly infectious viral illness that predominantly impacts children and can result in paralysis or, in severe cases, death [1]. Despite significant progress in global eradication efforts, Polio remains endemic in Pakistan and Afghanistan, continuing to challenge eradication efforts in these regions. The resurgence of polio in Pakistan in 2024 highlights not only the enduring threat of the disease but also the significant challenges in achieving full eradication. Despite relentless vaccination efforts, a combination of logistical difficulties and natural disasters has allowed the virus to persist and spread. In the year 2024, Pakistan reported 18 new cases of wild poliovirus type 1 (WPV1) till September [2], and there has been an alarming rise in WPV1-positive environmental samples from 126 in 2023 to 186 in 2024 [3]. These figures underscore the scale of the issue and the urgent need for a renewed focus on eradication

One of the primary barriers in recent years has been the COVID-19 pandemic, which severely disrupted routine immunization services [4]. During the pandemic, lockdowns, the reallocation of healthcare resources, and fear of visiting healthcare centers caused a significant reduction in vaccine coverage. The temporary suspension of polio campaigns meant that many children missed critical doses, leading to immunity gaps. These gaps refer to incomplete protection among children who received some, but not all, recommended doses of the polio vaccine. For instance, while children who received a dose of inactivated polio vaccine (IPV) may have developed systemic or humoral immunity, but they lack mucosal immunity, which is crucial for preventing virus replication and transmission in the intestines. As a result, these children could unknowingly contribute to silent transmission of poliovirus, as detected by environmental surveillance, even in the absence of clinical cases. As the pandemic subsided, these gaps contributed to the resurgence of polio cases, leaving thousands of children vulnerable to infection. In addition to the pandemic, natural disasters, particularly devastating floods, have further hindered efforts to eradicate polio [5]. The monsoon floods of 2022 displaced millions of people and destroyed critical infrastructure, cutting off healthcare access for weeks in some areas. Provinces like Balochistan and Sindh, already facing health system challenges, saw polio campaigns halted as the country focused on disaster relief [6]. Even in 2024, the lasting effects of these floods endure, as some regions remain inaccessible or lack basic healthcare services.

Before the pandemic, Pakistan’s polio immunization campaigns reached over 95% of the target population in high-risk districts, particularly in marginalized communities However, during the pandemic and subsequent floods, coverage dropped to as low as 65% in some areas, with Balochistan and Sindh provinces being the most affected. These regions have reported persistently low routine immunization rates even post-pandemic, reflecting systemic challenges in recovering from these disruptions. The decline underscores the urgent need to strengthen routine [7]. The continued increase in WPV1-positive samples suggests that widespread viral transmission is ongoing, particularly in areas hardest hit by these disasters. Erratic weather patterns, including increased rainfall and flooding, have worsened sanitation conditions [8], providing fertile ground for viral transmission. Contaminated water supplies, particularly in flood-prone regions, have facilitated the spread of poliovirus, which is transmitted through fecal-oral routes [9]. Poor sanitation exacerbates this risk, leading to the detection of viral samples in the environment even where no clinical cases have yet been reported. Political instability and security concerns are additional barriers that have impeded polio eradication efforts. In some parts of Pakistan, particularly in the tribal areas near the Afghanistan border, militant groups have targeted health workers. This violence has created a climate of fear, preventing vaccination teams from reaching many high-risk areas. Without adequate access to these regions, the virus is able to spread unchecked across borders, impacting children in both Pakistan and Afghanistan. Another key challenge is vaccine hesitancy, fueled by widespread misinformation and distrust of the healthcare system [10]. In some regions, parents refuse to vaccinate their children due to fear of the vaccine’s potential side effects or suspicion of foreign intervention [11]. In many regions of Pakistan, local beliefs and misinformation play a critical role in shaping public attitudes towards vaccines. Distrust in the healthcare system, often rooted in historical grievances, is further fueled by rumors spread via social media. For example, myths suggesting that polio vaccines lead to infertility have gained traction, creating widespread fear among parents. Engaging community leaders and religious scholars to counteract such narratives has shown promise in rebuilding trust. Involving trusted figures in advocacy campaigns and organizing community dialogue sessions can help address these concerns effectively and foster a positive attitude towards vaccination. These misconceptions, often spreading through social media and word of mouth, have allowed the virus to persist in communities that have otherwise had access to vaccination programs.

The worldwide risk posed by circulating vaccine-derived poliovirus (cVDPV) adds another layer of complexity to eradication efforts, In 2024, clinical 72 cases of cVDPV were confirmed globally till September, of which 68 were cVDPV2 and four were cVDPV1. Although many countries, including Algeria, Egypt, and Uganda, have detected cVDPV in environmental samples, no human cases have been reported.^2^ While Pakistan is still reporting clinical cases of cVDPV, countries like Algeria, Egypt, and Uganda have only detected the virus in wastewater samples without any associated human cases. This discrepancy can be attributed to robust routine immunization programs in these countries, which maintain high levels of population immunity. By achieving widespread vaccine coverage, these nations have significantly reduced the risk of poliovirus transmission, thus preventing outbreaks even when the virus is present in the environment. Additionally, effective sanitation infrastructure and public health surveillance in these countries play critical roles in mitigating the spread of the virus. These factors underscore the importance of strengthening routine immunization and sanitation measures in Pakistan to achieve similar outcomes. But the presence of cvdpv even in environmental samples however, highlights the need for continued vigilance and expanded vaccination efforts to prevent outbreaks.

Currently Pakistan’s outbreak response efforts have included targeted catch-up campaigns focusing on children under five years old, who receive multiple doses of oral polio vaccine (OPV) and, where feasible, inactivated polio vaccine (IPV). These campaigns, conducted in collaboration with international health organizations, aim to bridge immunity gaps caused by missed doses during the pandemic and natural disasters. For instance, in high-risk districts, door-to-door vaccination drives have been combined with community engagement activities to ensure better coverage. These efforts have delivered millions of doses, yet sustaining this momentum will require robust logistical planning and expanded outreach

To overcome the barriers to polio eradication, Pakistan must adopt a multifaceted strategy that addresses immediate challenges while ensuring long-term resilience. Vaccine hesitancy must be tackled through robust public health campaigns that build trust in vaccines, leveraging the influence of community and religious leaders to spread accurate information about vaccination benefits. Simultaneously, improving healthcare infrastructure and restoring services in flood-affected regions are critical to ensuring consistent vaccine availability. Enhanced collaboration with Afghanistan is also essential to combat cross-border transmission, allowing both countries to reach children in conflict-prone and remote areas. In addition to immediate measures, Pakistan must prioritize building resilient health systems capable of withstanding future disruptions from pandemics or natural disasters. This includes investing in healthcare infrastructure in vulnerable regions, enhancing emergency preparedness through disaster response frameworks, and strengthening routine immunization programs by integrating them with other essential healthcare services. Utilizing digital tools to track immunization coverage can help maintain progress even during crises. Furthermore, fostering community engagement and establishing partnerships across sectors such as water, sanitation, and education will create a holistic approach to addressing the underlying factors that enable poliovirus transmission, ensuring long term sustainable eradication efforts.

## Conclusion

The resurgence of polio in Pakistan serves as a crucial wake-up call, emphasizing the urgent need for sustained and coordinated action among health authorities, communities, and policymakers to combat this preventable disease effectively. By addressing the barriers of misinformation, natural disasters, and security threats, Pakistan can move closer to its goal of becoming polio-free. Additionally, improving flood resilience through infrastructure development, emergency preparedness, and intersectoral collaboration–particularly in water, sanitation, and healthcare–will be vital to mitigating the impact of increasingly extreme weather events driven by climate change. The time to act is now, before more children are affected by this preventable disease.
